# Biosensor Applications of MAPLE Deposited Lipase

**DOI:** 10.3390/bios4040329

**Published:** 2014-10-03

**Authors:** Valeria Califano, Francesco Bloisi, Antonio Aronne, Stefania Federici, Libera Nasti, Laura E. Depero, Luciano R. M. Vicari

**Affiliations:** 1 Istituto Motori—CNR, via G. Marconi 8, 80125 Napoli, Italy; 2 Department of Physics, University of Naples “Federico II”, Complesso Universitario di Monte Sant’Angelo, via Cintia, 80126 Napoli, Italy; E-Mails: bloisi@na.infn.it (F.B.); lidar@na.infn.it (L.N.); vicari@unina.it (L.R.M.V.); 3 SPIN—CNR, c/o Dipartimento di Fisica, Complesso Universitario di Monte Sant’Angelo, via Cintia, 80126 Napoli, Italy; 4 Department of Chemical Engineering, Materials and Industrial Production, University of Naples “Federico II”, Piazzale V. Tecchio 80, 80126 Napoli, Italy; E-Mail: anaronne@unina.it; 5 DIMI—Dipartimento di Ingegneria Meccanica ed Industriale, University of Brescia, via Branze 38, 25123 Brescia, Italy; E-Mails: stefania.federici@unibs.it (S.F.); laura.depero@unibs.it (L.E.D.)

**Keywords:** MAPLE, lipase, biosensors

## Abstract

Matrix Assisted Pulsed Laser Evaporation (MAPLE) is a thin film deposition technique derived from Pulsed Laser Deposition (PLD) for deposition of delicate (polymers, complex biological molecules, *etc*.) materials in undamaged form. The main difference of MAPLE technique with respect to PLD is the target: it is a frozen solution or suspension of the (guest) molecules to be deposited in a volatile substance (matrix). Since laser beam energy is mainly absorbed by the matrix, damages to the delicate guest molecules are avoided, or at least reduced. Lipase, an enzyme catalyzing reactions borne by triglycerides, has been used in biosensors for detection of β-hydroxyacid esters and triglycerides in blood serum. Enzymes immobilization on a substrate is therefore required. In this paper we show that it is possible, using MAPLE technique, to deposit lipase on a substrate, as shown by AFM observation, preserving its conformational structure, as shown by FTIR analysis.

## 1. Introduction

MAPLE (Matrix Assisted Pulsed Laser Evaporation) [1] is a thin film deposition technique derived from PLD (Pulsed Laser Deposition). Despite that PLD (a pulsed laser beam focused, inside a vacuum chamber, on the material to be deposited, in form of a solid target, vaporizes it, as a plasma plume, allowing deposition on the substrate placed in front of the target) is a well established technique nowadays for the deposition of high quality thin films [2], it fails in applications where polymers, biological materials or, in general, delicate and large molecules are involved [3].

**Figure 1:**
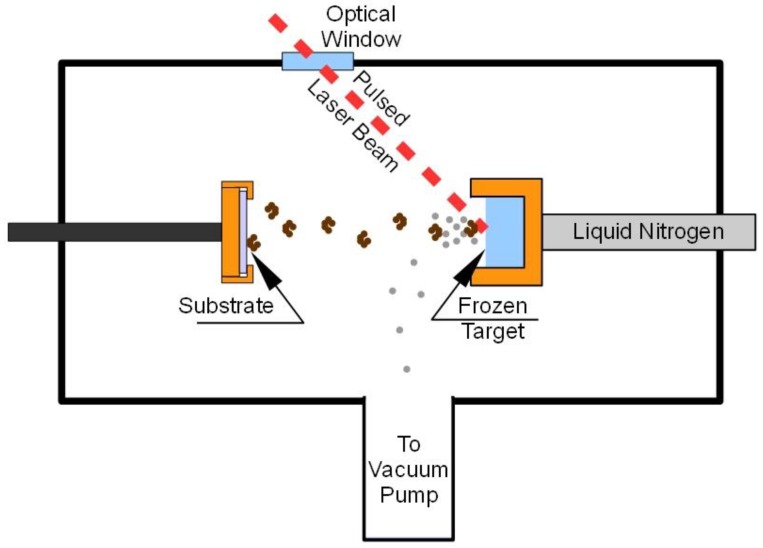
Typical MAPLE deposition system.

The main difference of MAPLE with respect to PLD technique is the target: it is a frozen (usually by immersion or just thermal contact with liquid nitrogen) solution or suspension of the molecules to be deposited (guest) in a volatile substance (matrix) [4]. A pulsed laser beam (Figure 1) impinging onto the target is used to induce “evaporation” of the matrix together with the guest molecules. Since the deposition is carried out within a vacuum chamber, the light matrix molecules are taken away by the vacuum pump system and only guest molecules reach the substrate placed in front of the target. The “laser ablation” is therefore replaced by a more gentle “laser evaporation” and, despite the presence of a “solvent” in the target, the MAPLE deposition is essentially “solvent-free”. The simple evaporation model initially assumed for MAPLE depositions has been shown to be inadequate in describing the complex physical process involved in the ablation of such a composite target, but in any case the presence of the matrix avoids or at least reduces the damage of the guest molecules induced by laser radiation.

Good results have been reported by many authors, in recent years, for an increasing number of applications [5,6,7,8,9] requiring the deposition of organic molecules, biomaterials or polymers on a substrate. Proteins and enzymes [10,11,12,13] have also been deposited using MAPLE technique.

Enzymes are widely involved as sensing elements in biosensors [14,15] combining a thin layer of insolubilized (immobilized) enzyme with an electrochemical probe.

Lipases [16] are an important enzymes found in almost all biological systems. Their physiological role is to catalyze the hydrolysis of triacylglycerol to glycerol and fatty acids [17]. This allows the use of lipase miniaturized silicon biosensor for detection of triglycerides (TG) in blood serum [18].

In general, lipases are able to catalyze hydrolysis involving carboxylic ester bonds. Some lipases can do that with a high enantiomeric specificity and have been studied as a biosensor for enantioselective analysis of β-hydroxy acid esters for pharmaceutical purposes [19].

**Figure 2:**
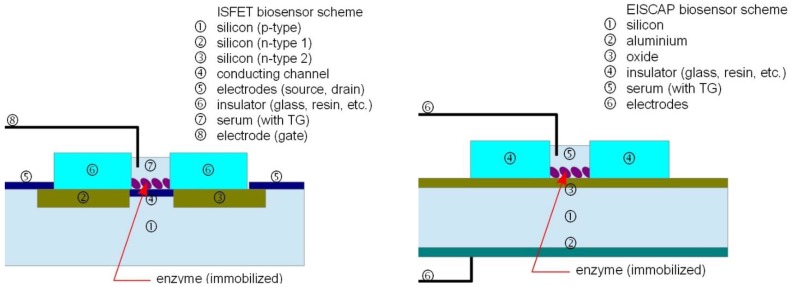
Typical TG biosensors realized as ISFET or EISCAP devices.

Typical TG biosensors using an enzymatic reaction are realized as ion sensitive field effect transistors (ISFET) or electrolyte insulator semiconductor capacitor (EISCAP) devices (Figure 2), both having some advantages: the first is characterized by great simplicity of design and of readout (the drain current is a function of the pH) while the second is more robust and of easy fabrication while readout is obtained examining the capacity-voltage (C-V) characteristics (modulated by the pH of the electrolyte). The TG is hydrolyzed by the lipase, thus changing the pH of the electrolyte (blood serum) affecting the value of the drain current (in an ISFET sensor) or shifting the C-V plot (in an EISCAP sensor). The enzyme for hydrolysis is integrated in the sensor by immobilization on the silicon substrate.

To get an efficient technique for lipase immobilization is a rather difficult task, especially because until today there were not systematic theories able to guide fundamental choices such as the immobilization methodology and the support.

Enzymes can be immobilized by several techniques, classified in four categories: physical adsorption onto an insoluble carrier [17], entrapment within a matrix [20], cross-linking [21] and covalent bonding [22] to an insoluble support.

Physical absorption onto insoluble carriers is maybe the easiest and cheapest way to immobilize lipases. The method relies on physical interactions between the enzyme and the carrier’s surface (mainly Van der Waals forces, hydrogen bonding and hydrophobic interactions) and is realized by keeping in contact a concentrated solution of lipase with the support. This procedure is conservative with respect to lipase since it does not involves chemical modification of the enzyme and is conducted under mild conditions. It is also often possible to recover the support, to reduce costs. Despite the straightforwardness of the process, some critical issues have to be addressed.

Actually, the adsorption of lipase onto the carrier depends on several parameters. First, the pH at which the adsorption is conducted has to be close to the isoelectric point of the enzyme (pH at which the total positive and negative charges on the amino-acid residues are the same, so the molecule is overall neutral) to maximize the loading of adsorbed lipase, since at isoelectric point the solubility of lipase decreases. Values of isoelectric pH of lipases are often acidic. For example lipase from Candida Rugosa, used in this study, has an isoelectric point of 4.5 [23].

To decrease the solubility of lipase in water, hence maximizing adsorption, addition of water-miscible solvent during the immobilization process or vaporization of the water under reduced pressure have been used [24]. However, the problem of loading is not trivial. The effect of lipase loading was studied [25] and a bell-shaped curve was obtained, with a maximum catalytic efficiency at a load corresponding to 10% of the carrier (Accurel EP100) saturation value. At low loadings the lipase molecules attempt to maximize its contact with the excess area available and hence they undergo important conformational changes. At high loadings, the efficiency drops as a result of diffusion limitations. Similar results were obtained with polypropylene as support material [26].

In second instance, the adsorption of proteins on solid surfaces can induce structural changes that can affect the entire macromolecule, hence its catalytic activity, which is tightly bound to the enzyme tertiary structure. The immobilization should not significantly reduce the activity and stability of the enzyme, but on the contrary improve them. There is no rule that allows predicting the effect of immobilization on the activity and stability of the enzyme: it may result in either activation or inhibition of the biocatalyst [27]. The nature of the support is very important in determining the efficiency of the immobilization, as it can influence the conformation of the enzyme during adsorption. Generally hydrophobic surfaces are better materials for immobilization. Actually, most lipases display an increase in catalytic activity when immobilized on these supports. This characteristic has been shown to be associated with conformational changes in the enzyme upon adsorption, creating an open, substrate-accessible active site. Thus, lipases recognize hydrophobic surfaces similar to those of their natural substrates and they undergo interfacial activation during immobilization [28].

For all the above procedures the immobilization efficiency, especially for high enzyme loading, is mainly related to the ability to preserve the native conformation of the enzyme. Although there are several immobilization protocols, the design of new protocols that may permit improvement to the enzyme properties during immobilization is still an exciting goal [29] and MAPLE technique is a good candidate for deposition of lipase preserving its active configuration.

In this paper we show that it is possible, using MAPLE technique, to deposit lipase on a substrate, as shown by AFM observation, preserving its conformational structure, as shown by FTIR analysis. MAPLE technique is therefore a promising technique for immobilization of lipase in biosensors production. We do not present or discuss a particular biosensor but we suggest the use of a novel technique for enzymes immobilization in biosensors and we demonstrate its feasibility presenting the results of preliminary tests.

## 2. Experimental Section

Candida Rugosa Lipase (CRL) type VII was obtained by Sigma-Aldrich, chosen for its positional aspecificity (the capability of acting on all glyceride bonds, independently from their position). Since MAPLE deposition is carried out starting form a solution, non-buffered demineralized water was used as solvent.

The MAPLE [4] deposition technique is essentially based on using for laser ablation a target obtained as a frozen solution or suspension of the guest material (will be deposited on the substrate) in a matrix (will be pumped away during deposition). Such approach allows a guest extraction technique more gentle than laser ablation (laser energy is mainly used to obtain matrix evaporation) making possible the deposition of undamaged soft (e.g., polymeric, organic, bio) materials.

MAPLE generally allows a high control of the characteristics of the deposited film, even better than PLD since film growth is generally much lower and the deposition process is also influenced by other parameters as solvent properties and concentration, substrate and target temperature, and so on. Even if in the present application the morphology of the surface is not of primary interest, both rough and smooth layers can be produced depending on the deposition conditions.

Therefore MAPLE shows several advantages with respect to other deposition techniques, such as ink-jetting or drop-casting. In first place it is a dry technique and therefore allows deposition on substrates that would be damaged by the solvent. Moreover, once the deposition parameters have been optimized for the specific application, it allows the production of better quality films with higher order parameters.

As previously stated, the target is frozen by placing it in thermal contact with liquid nitrogen contained in an external reservoir. A pulsed laser beam impinging on the target removes from its surface both the matrix and guest molecules, but only the latter reaches the substrate, placed in front of the target. The presence of the matrix (the solvent) eliminates or at least minimizes the possible photochemical damage of the delicate molecules due to laser irradiation.

A freshly prepared 2wt% lipase in water solution is placed in the target holder, placed in a vacuum chamber (initially at ambient pressure). Its temperature is then lowered to about −123 °C by filling a reservoir in thermal contact with the target holder with liquid nitrogen. The vacuum system is then operated in order to reduce the chamber pressure to about 10^−4^ Pa. The substrate is placed in front of the target at a distance of about 1 cm and the Nd:YAG pulsed laser is operated at its fundamental wavelength (1064 nm).

The target to substrate distance determines the morphology of the deposition: increasing the distance enhances the uniformity of the film while decreasing the distance increases the amount of deposited material. The first approach is generally useful for organic electronics applications. In this paper we have chosen to privilege the second one since we are mainly interested in demonstrating the feasibility of the use of MAPLE technique for biosensor realization, and in particular the ability of the lipase to maintain its features after having undergone both the freezing and the laser extraction processes. Within other parameters concerning the MAPLE deposition, it is important to take into account the wavelength (or the corresponding photon energy) of the pulsed laser beam used for target ablation/evaporation, as clearly discussed in [30]. It must be absorbed by matrix (most common solvents absorb UV radiation while are almost transparent to IR) but must also be chosen in order to avoid damages to guest molecules (IR radiation corresponds to lower-energy photons with respect to UV). 

The Nd:YAG laser equipped with Multiple (Second, Third, *etc*.) Harmonic Generation (nHG) device can be operated at different wavelengths from UV to IR (266 nm, 355 nm, 532 nm, 1064 nm). The deposition wavelength has been chosen at IR wavelength for minimizing possible photochemical decomposition of the lipase, since it has a large absorption band, centered at 280 nm [31], in the UV region. The laser is operated in Q-switched mode and the pulse duration is 10 ns.

The laser beam, impinging on the target at an angle of 45 deg, is slightly focused to an ellipsoidal area of about 1.0 mm by 1.4 mm. In order to avoid drilling, the target is moved by a computerized 2D translation system so that the beam scans (one or more times, depending on the total number of pulses) an area of about 1.5 cm2. Several combinations of laser beam parameters (pulse repetition rate, fluence, number of pulses) can be used as discussed elsewhere [32]. Here, the laser beam has been operated at 4 pulses/s, 526 mJ/pulse, for 29,720 pulses. 

The energy per pulse value has been chosen, according to preliminary tests, slightly higher than the minimum required to get a deposition. In order to reduce both the required energy per pulse and the corresponding photon energy, since the matrix is frozen water, it would be possible to use the 2940 nm wavelength radiation from an Er:YAG laser, but in our case the guest material (lipase) has a wide absorption peak near 3400 cm−1, as shown by FTIR spectra, thus discouraging the use of such laser wavelength.

MAPLE depositions have been carried out on KBr pellets having 13 mm diameter for FTIR analysis and on glass substrate for AFM image examination.

The FTIR spectrometer was equipped with a DTGS KBr (deuterated triglycine sulphate with potassium bromide windows) detector and spectra were recorded with a spectral resolution of 2 cm^−1^, in the range 4000–400 cm^−1^, and each spectrum was obtained as an average of 32 scans, corrected for the spectrum of the blank KBr pellet.

## 3. Results and Discussion

**Figure 3:**
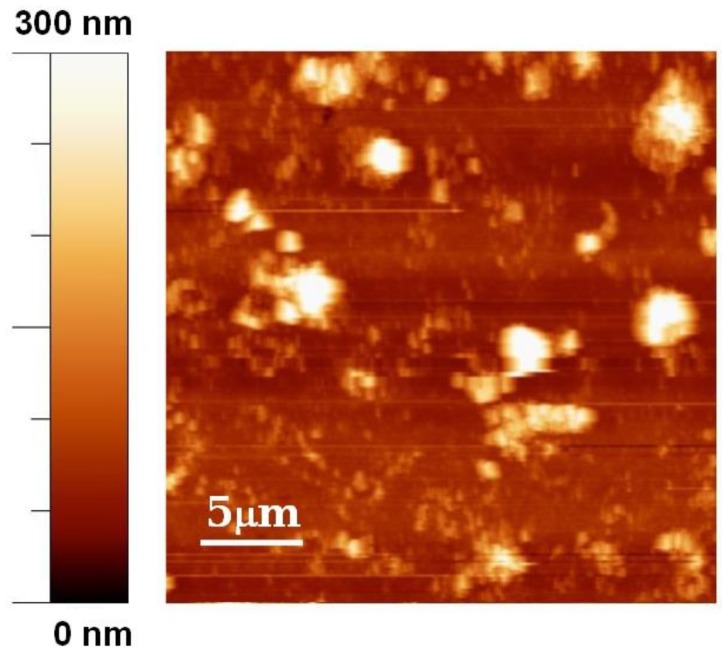
AFM image of lipase deposited by MAPLE on a glass substrate.

The AFM image (Figure 3) shows the presence three-dimensional structures with sizes of order of magnitude of about one micron. The presence of complex surface morphologies is not uncommon in depositions carried out with MAPLE technique [33,34,35,36] and is due to the ejection and deposition of droplets generated in the process of the explosive disintegration of the overheated matrix. However, the morphology of the film is not expected to invalidate the functionality of the biosensor.

**Figure 4:**
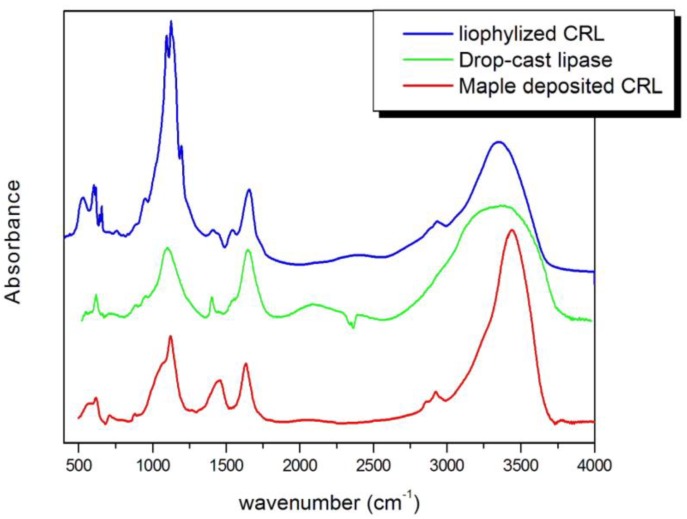
The FTIR spectra of lipase after MAPLE deposition procedure (MAPLE-CRL) compared with same spectra from lipase before deposition (lyophilized CRL) and drop-cast lipase.

Infrared spectroscopy has been used to study the secondary structure of proteins both in water solution [37,38] and in the solid state [39,40], so we use this technique in order to verify that Candida Rugosa Lipase (CRL) has been deposited in undamaged form, FTIR spectra of lipase deposited with MAPLE technique (MAPLE-CRL spectra) on KBr pellets have been compared with analogous spectra obtained from pure lipase (free-CRL spectra). The latter have been obtained taking the spectra of special KBr/lipase pellets made using 4.0 mg of lyophilized unprocessed lipase (free-CRL) mixed with 200 mg of KBr. The spectra are shown in Figure 4 in the wavenumber range 400–4000 cm^−1^. The polypeptide and protein repeat units give rise to nine characteristic IR absorption bands, namely amide A, B and I-VII [41]. Of these, amide I and II are the most prominent vibrational bands of the protein backbone. The amide I band (1600 to 1700 cm^−1^), mainly due to C=O stretching vibrations, is the most sensitive spectral region to the protein secondary structural components. The amide II (1480 to 1580 cm^−1^) band, related with N-H bending and C-N stretching vibrations, shows much less conformational sensitivity than its amide I counterpart. Other amide vibrational bands (amide III 1200 to 1300 cm^−1^, amide IV–VI 537–800 cm^−1^, amide A-B 3100–3300 cm^−1^) are very complex depending on the details of the force field, the nature of the side chains and hydrogen bonding. Spectral analysis of MAPLE deposited lipase shows the presence of all the bands attributed to the peptide bond vibration together with the presence, around 1100 cm^−1^ of the carbohydrate band. Even if a significant increase of the intensity of the amide II' peak (1450 cm^−1^, C-N component of the amide II band) and a shift of a lower wavenumber of the amide I band indicate some conformational rearrangement of the enzyme, there is evidence of preservation of enzyme structure after MAPLE deposition. In particular, the displacement of amide I towards lower wavenumbers can be indicative of aggregation phenomena taking place before (solution preparation and freezing) or during (laser impact, dehydration during plume expansion) MAPLE deposition. In fact, amide I is due to the overlapping of stretching vibration of C=O bond in different environments: α-helices, β-sheets, turns, disordered regions and intermolecular aggregate β-strands. Aggregation is a common phenomenon in proteins and can highly influence their activity, hence their efficiency as reacting element in a biosensor architecture. The IR absorption of aggregated strands is the lower wavenumber component of amide I (1620 cm^−1^) and is the probable reason for its shift.

Furthermore, the band between 900 and 1200 cm^−1^, which shows a major difference in the two lipases, is not associated with the protein backbone, but with sugar chains bound to some protein sites, and depends on the degree of hydration of the protein, which is obviously different between lyophilized and MAPLE-deposited lipase. A demonstration of this statement can be seen on the FTIR spectra of Figure 4, where is reported the FTIR curve of drop-cast lipase, deriving from a drop of lipase aqueous solution spilled on a KBr pellet and air-dried.

## 4. Conclusions

The enzyme lipase, found in almost all biological systems where it acts as a catalyst in the hydrolysis of triacylglycerol to glycerol and fatty acids, is used in biosensors for detection of β-hydroxyacid esters and triglycerides in blood serum. An important issue is its deposition/immobilization on a substrate. We have shown that MAPLE technique allows deposition of lipase and that the whole process (freezing, laser irradiation, vacuum drying, and impact on substrate) preserve the conformational characteristics of the enzyme, as shown by FTIR analysis, a technique used to study the secondary structure of proteins both in water solution and in solid state.
